# Attenuation of visceral nociception by α-bisabolol in mice: investigation of mechanisms

**DOI:** 10.1186/2191-2858-2-18

**Published:** 2012-05-21

**Authors:** Gerlânia de Oliveira Leite, Cícera Norma Fernandes, Irwin Rose Alencar de Menezes, José Galberto Martins da Costa, Adriana Rolim Campos

**Affiliations:** 1 Departamento de Química Biológica, Universidade Regional do Cariri, Crato, CE 63105-000, Brazil; 2 Vice-Reitoria de Pesquisa e Pós-Graduação, Universidade de Fortaleza, Av. Washington Soares, 1321, Fortaleza, Ceará, CEP 60811-905, Brazil

**Keywords:** α-bisabolol, Visceral nociception, Mechanisms

## Abstract

**Background:**

We previously described the visceral antinociceptive property of α-bisabolol (BISA) in mouse models of visceral nociception induced by cyclophosphamide and mustard oil (MO). This study examined the effect of BISA in mouse models of visceral nociception induced by acetic acid, capsaicin, formalin, and the contribution of the nitric oxide system, α_2_, K_ATP_, 5-HT_3_ and TRPV1 receptors to the effect of BISA on MO-evoked nociceptive behaviors. Mice were pretreated orally with BISA (50, 100 and 200 mg/kg) or vehicle, and the pain-related behavioral responses to intraperitoneal administration of acetic acid or intracolonic injection of MO were analyzed.

**Results:**

BISA significantly suppressed the nociceptive behaviors in a dose-unrelated manner. The antinociceptive effect of BISA (50 mg/kg) was show to be glibenclamide resistant, but it was not blocked by pretreatment with the other antagonists tested. In the open-field test that detects sedative or motor abnormality, mice received 50 mg/kg BISA did not show any per se influence in ambulation frequency.

**Conclusions:**

However, their precise antinociceptive mechanisms of action have not been determined.

## Background

Essential oils are natural products that exhibit a variety of biological properties such as analgesic, anticonvulsant, and anxiolytic ones. Such effects are often attributed to the presence of monoterpenes, which are the major chemical components of these oils [1]. Sesquiterpenes, such zerumbone [2], budlein A [3], polygodial [4], and lapidin [5], have been shown to present antinociceptive properties.

Recent studies demonstrated that the sesquiterpene α-bisabolol (BISA) presents antitumor [6], peripheral nervous blocker [7], gastroprotective [8], leishimanicidal [9], antioxidant [10], mutagenic and antimutagenic [11], and wound healing [12] properties. There are also evidences showing that BISA reduced visceral nociceptive pain evoked by intracolonic administration of mustard oil (MO) as well as that induced by intraperitoneal injection of cyclophosphamide, possibly involving the inhibition of sensitization of primary afferent neuron terminals by prostaglandins [13].

However, the mechanisms underlying these antinociceptive properties of BISA are not well understood.

## Methods

### Animals

Male Swiss albino mice (20–25 g) obtained from the Central Animal House of Regional University of Cariri were used. They were housed in environmentally controlled conditions (22°C, 12-h light–dark cycle), with free access to standard pellet diet (Purina, São Paulo, Brazil) and water. Animals were kept in cages with raised floors to prevent coprophagy. Before the visceral antinociceptive assays, they were fasted over a period of 15 h and were habituated to the test environment for 2 h before the experimentation. The experimental protocols were in accordance with the ethical guidelines of National Institute of Health, Bethesda, USA. The studies were performed in a blinded manner.

### Acetic acid-induced visceral nociception

Abdominal constrictions were induced by intraperitoneal injection of acetic acid (0.6%). The animals were pretreated with BISA (50, 100 or 200 mg/kg, p.o.) or vehicle (2% Tween 80, 10 mL/kg, p.o.) 60-min prior to acetic acid injection. After the challenge, each mouse was placed in a separate glass funnel and the number of contractions of the abdominal muscles, together with stretching, was cumulatively counted over a period of 20 min. Antinociceptive activity was expressed as the reduction in the number of abdominal contractions, comparing the control animals with the mice pretreated with BISA.

### Capsaicin-induced visceral nociception

Male mice divided into groups of eight in each were pretreated with the vehicle (2% Tween 80 in distillated water, 10 mL/kg, p.o.) or BISA (50, 100 or 200 mg/kg, p.o.). 1 h after oral and 30 min following systemic treatments, capsaicin (0.3% in a solution of PBS:Tween 80:ethanol (8:1:1)) was instilled into the colon (50 μL/animal) using a fine cannula (1.6-mm external diameter), 4-cm far from the anal sphincter. Solid petroleum jelly was applied onto the perianal region to avoid local nerve stimulation. The animals were then observed during a 30-min period for the spontaneous visceral pain-related behaviors (licking the upper abdomen, abdominal contortion and retraction, squashing the abdomen against the floor). A normal group, that received only saline intracolonically, was also included.

### Formalin-induced visceral nociception

Male mice divided into groups of eight in each were pretreated with the vehicle (2% Tween 80 in distillated water, 10 mL/kg, p.o.) or BISA (50, 100 or 200 mg/kg, p.o.). 1 h after oral and 30 min following systemic treatments, formalin (10%) was instilled into the colon (10 μL/animal) using a fine cannula (1.6-mm external diameter), 4-cm far from the anal sphincter. Solid petroleum jelly was applied onto the perianal region to avoid local nerve stimulation. The animals were then observed during a 60-min period for the spontaneous visceral pain-related behaviors (licking the upper abdomen, abdominal contortion and retraction, squashing the abdomen against the floor). A normal group, that received only saline intracolonically, was also included.

### MO-induced visceral nociception

To assess the antinociceptive effect of BISA against the MO-induced visceral nociception, mice in groups (*n* = 8) were orally treated with BISA (50, 100 or 200 mg/kg, p.o.) or vehicle (2% Tween 80 in distillated water, 10 mL/kg, p.o.) 1 h before the intracolonic administration of MO (0.75% in saline 0.9%, 50 μL/animal). A group of normal control received a similar dose of saline (10 mL/kg). Immediately following the intracolonic MO or saline administration, the mice were observed for the total number of nociceptive behaviors (licking the upper abdomen, stretching the abdomen, squashing the abdomen against the floor and retraction of the abdomen characterized for an arched position), for a 20-min period.

In order to verify the possible involvement of nitrergic, noradrenergic, K_ATP_, 5-HT_3_, and TRPV1 receptors in the effect of BISA, the animals were treated with L-NAME (10 mg/kg, i.p), yohimbine (2 mg/kg, i.p), glibenclamide (5 mg/kg, i.p), ondansetron (10 mg/kg, i.p) or ruthenium red (3 mg/kg, s.c.), 30 min before the administration of BISA (50 mg/kg).

### Locomotor activity (open-field test)

The open-field area was made of acrylic (transparent walls and black floor: 30 × 30 × 15 cm^2^) divided in nine squares of equal area. Four groups of animals (*n* = 6) were used. While groups 1 and 2 were treated, respectively, with vehicle (2% Tween 80, 10 mL/kg, p.o.) and BISA (50 mg/kg, p.o.), groups 3 and 4 received vehicle + MO (0.75%, 50 μL) or BISA (50 mg/kg, p.o.) + MO. The MO was given by an intracolonic route 90 following the vehicle or BISA. The numbers of squares each animal crossed with the four paws were noted during a 4-min period.

### Statistical analysis

The results are expressed as mean ± SEM from six or eight mice per group. For statistical analysis, ANOVA followed by Tukey’s or Student–Newman–Keul’s post hoc test, as appropriate, were used. A *p* < 0.05 was considered statistically significant.

## Results and discussion

### Effect of BISA on acetic acid, capsaicin, formalin and MO-induced visceral pain in mice

Table 1 shows the antinociceptive effect of BISA in acetic acid, capsaicin, formalin and MO. Intraperitoneal application of acetic acid (0.6%) provoked a significant increase in abdominal constrictions when compared with saline-treated normal control. In groups pretreated with BISA (50, 100 and 200 mg/kg, p.o.), acetic acid-induced abdominal constrictions were significantly inhibited in a dose-unrelated manner. Intracolonic application of capsaicin (CAP 0.3%), MO (MO 0.75%), or formalin (10%) provoked a significant increase in spontaneous pain-related behaviors when compared with saline-treated normal controls (Table 1). In groups pretreated with BISA, capsaicin, formalin and MO-induced nociceptive behaviors were significantly inhibited. L-NAME, yohimbine, glibenclamide, ondansetron, or ruthenium red failed to revert the antinociceptive effect of BISA (Tables 2, 3 and 4). Table 5 shows the effects of orally administered BISA on behavior in open-field test in mice. At the dose tested (50-mg/kg), BISA showed no significant influence on the number of crossings in open-field test.

**Table 1:** Antinociceptive effect of BISA in capsaicin, formalin, MO, and acetic acid-induced visceral nociception in mice

**Group**	**Dose (mg/kg)**	**Specific behaviors**			
		**Capsaicin**	**Formalin**	**MO**	**Acetic acid**
Normal	-	10.25 ± 6.21**	198.60 ± 47.87**	21.57 ± 7.13***	-
Vehicle	-	84.38 ± 12.99	344.60 ± 50.21	107.80 ± 25.01	48.63 ± 10.81
BISA	50	87.00 ± 19.30	103.00 ± 16.21***	21.67 ± 7.99***	10.50 ± 2.94***
	100	31.00 ± 13.11**	140.40 ± 25.51***	33.83 ± 10.28***	8.50 ± 3.20***
	200	15.33 ± 10.82**	75.13 ± 13.44***	27.17 ± 11.05***	8.43 ± 2.57***

Values represent the mean ± SEM of pain-related behaviors (licking of abdomen, stretching, abdominal retractions). **p < 0.01 and ***p < 0.001 versus vehicle (ANOVA, Student-Newman-Keul’s test).

**Table 2:** Effect of yohimbine, ondansetron, L-NAME, glibenclamide and ruthenium red against MO-induced visceral pain in mice

**Group**	**Dose (mg/kg)**	**Specific behaviors**
Normal	-	18.14 ± 3.44*
Vehicle	-	44.17 ± 5.59
Yohimbine	2	22.28 ± 3.53*
Ondansetron	0.5	44.75 ± 5.89
L-NAME	20	27.13 ± 3.74
Glibenclamide	5	40.88 ± 8.39
Ruthenium red	0.3	29.25 ± 4.98

Values represent the mean ± SEM of pain-related behaviors (licking of abdomen, stretching, abdominal retractions). *p < 0.05 versus vehicle (ANOVA, Student-Newman-Keul’s test).

**Table 3:** **Effect of α**_**2**_**-adrenoceptor antagonism on the antinociceptive effect of BISA in MO test**

**Group**	**Dose (mg/kg)**	**Specific behaviors**
Normal	-	4.25 ± 1.77***
Vehicle		28.50 ± 4.00
BISA	50	1.29 ± 0.75***
BISA + yohibmine	50 + 2	5.75 ± 1.77***

Values represent the mean ± S.E.M. of pain-related behaviors (licking of abdomen, stretching, abdominal retractions). ***p < 0.001 *vs* Vehicle (ANOVA, Student-Newman-Keul’s test).

**Table 4:** **Evaluation of involvement of serotonergic, nitrergic, K**_**ATP**_**channels, and TRPV1 receptors in the antinociceptive effect of BISA in MO test**

**Group**	**Dose (mg/kg)**	**Specific behaviors**
Normal	-	88.25 ± 18.59**
Vehicle	-	181.90 ± 24.07
BISA	50	115.70 ± 25.33*
BISA + Ondansetron	50 + 0.5	57.13 ± 11.07***
BISA + L-NAME	50 + 20	31.25 ± 8.64***
BISA + glibenclamide	50 + 5	86.57 ± 15.61*
BISA + ruthenium red	50 + 3	61.71 ± 8.41**

Values represent the mean ± SEM of pain-related behaviors (licking of abdomen, stretching, abdominal retractions). *p < 0.05, **p < 0.01 and ***p < 0.001 versus vehicle (ANOVA, Student-Newman-Keul’s test).

**Table 5:** Effect of BISA on mouse behavior in open-field test

**Group**	**Number of crossings**
Vehicle	23.50 ± 5.04
BISA	13.57 ± 3.22
Vehicle + MO	31.86 ± 5.06
BISA + MO	19.00 ± 5.27

Mice were pre-treated with vehicle (2% Tween 80 in distilled water, *p.o.*) or BISA 50 mg/kg (*p.o.*), before intracolonic instillation of saline or MO (0.75%, 50 μL). Data represent mean ± SEM (n = 8). Data represent the mean ± SEM (n = 8). ANOVA, Student-Newman-Keul’s test).

In this study, we observed that the acetic acid, capsaicin, formalin, and MO-evoked nociceptive pain behaviors were significantly attenuated in mice pretreated with BISA, a natural sesquiterpenoid isolated from the different essential oils. It has been well established that many algogenic substances induce nociceptive pain-related behaviors in rodents following intraperitoneal or intracolonic application, and several reports reveal that naturally occurring compounds, like terpenoids, unsaturated dialdehydes and phenolic ketones can suppress these behaviors [14].

Many studies use acetic acid-induced effects as a model of visceral nociception but it lacks specificy [15]. Capsaicin, the pungent ingredient of red peppers applied topically or injected into the skin of humans or experimental animals, is known to stimulate the vanilloid receptor (Transient Receptor Potential cation channel V1 or TRPV1) located on polymodal C-fibers, but also in other tissues and initiates a complex cascade of events, including neuronal excitation and release of pro-inflammatory mediators, desensitization of receptor, and neuronal toxicity [16,17]. The intracolonic instillation of formalin via the anus evokes differentiated behaviors, which reflect visceral nociception. All types of behavior were dose dependently inhibited by morphine, indicating that they are pain-related [18]. As shown before, BISA could significantly suppress the nociception-related behaviors against MO-induced visceral nociception [13].

Visceral pain is a prominent form of pain in many clinical conditions [15]. The results obtained in the acetic acid-, capsaicin-, formalin-, and MO-induced test models of visceral nociception are shown in Table 1. In vehicle-treated control groups of mice, acetic acid-, capsaicin-, formalin-, and MO-induced spontaneous nociception-related behaviors when compared with respective saline-treated control groups. In groups pretreated with BISA, the nociceptive behaviors induced by algogenic substances were significantly inhibited in a dose-independent manner.

In the search of the possible action mechanism of BISA, we used the acute model of visceral nociception induced by intracolonic instillation of MO, which has disease relevancy to human irritable bowel syndrome [18]. The sesquiterpene BISA could significantly suppress the pain-related behaviors against MO-induced visceral nociception, possibly regulating the functioning of primary afferent fibers. Visceral afferents express a wide range of membrane receptors (including vanilloid receptors, TRPV1) to chemical stimuli, which are involved in sensory signaling from the gut to the central nervous system [19]. When animals were pretreated with BISA and ruthenium red (a non-competitive antagonist of TRPV1) in combination, there was additive antinociception in MO test. Also, BISA 50 mg/kg failed to inhibit the visceral nociception induced by capsaicin. These observations suggest that BISA does not act as a TRPV1 agonist but may possibly induce a modulatory influence on vanilloid-receptors, which needs further clarification.

Previous studies have shown that MO can induce acute colitis and BISA has been reported to exert antiinflammatory action [13,20]. Therefore, it is reasonable to assume that BISA suppresses the inflammatory pain. The α2-adrenoceptor agonist has been shown to induce antinociceptive effect in the experimental model of formalin-induced colitis in rats and reduce visceral hypersensitivity in clinical settings [21,22]. Therefore, a possible involvement of α2-adrenoceptors in the antinociceptive effect of BISA in MO-model of visceral pain, using the antagonist yohimbine was investigated. Yohimbine could not reverse the antinociception produced by BISA, suggesting that α2-adrenoceptors play no role.

K_ATP_ channel openers induce cell hyperpolarization, decrease the intracellular Ca^2+^ level and neurotransmitter release (calcitonin gene-related peptide and substance P), that may account for antinociception [23,24]. To verify such a possibility, we examined the effect of glibenclamide, a blocker of K_ATP_ channels on BISA antinociception. Pretreatment of mice with glibenclamide in combination with BISA showed no alteration. When mice were pretreated with L-NAME, a nitric oxide synthase inhibitor and the 5-HT_3_ antagonist, ondansetron, apparently a potentiated/additive response which needs further analysis through an isobolographic study.

Drugs that impair motor activity or induce sedation may give false-positive/negative results in nociceptive tests. We therefore sought to verify such effects of BISA on open-field test that detects motor incoordination [25]. The sesquiterpene failed to alter significantly the ambulation in open-field test. This result indicates that BISA exerts analgesia without causing neurological or muscular deficits.

## Conclusions

Although BISA efficient diminished the acetic acid, capsaicin, formalin, and MO-evoked pain-related behaviors, its mechanism is unclear from this study and future studies are needed to verify how the sesquiterpene exerts its antinociceptive action.

## Competing interests

The authors declare that they have no competing interest.

## Authors’ contributions

GOL carried out the antinociceptive studies. CNF carried out the antinociceptive studies . IRAM carried out the antinociceptive studies. JGMC carried out the antinociceptive studies. ARC carried out the antinociceptive studies and draft the manuscript. All authors read and approved the final manuscript.
